# Superanomalous skin-effect and enhanced absorption of light scattered on conductive media

**DOI:** 10.1038/s41598-023-31478-y

**Published:** 2023-03-29

**Authors:** A. Vagov, I. A. Larkin, M. D. Croitoru, V. M. Axt

**Affiliations:** 1 Theoretische Physik III, Universität Bayreuth, 95440 Bayreuth, Germany; 2 Institute of Microelectronics Technology, Russian Academy of Sciences, 142432 Chernogolovka, Russia; 3 Universidade Federal de Pernambuco, Recife, PE 50670-901 Brazil; 4 HSE University, 101000 Moscow, Russia

**Keywords:** Surfaces, interfaces and thin films, Nanophotonics and plasmonics, Nanophotonics and plasmonics, Spectrophotometry, Nanophotonics and plasmonics, Condensed-matter physics, Plasma physics

## Abstract

Light scattering spectroscopy is a powerful tool for studying various media, but interpretation of its results requires a detailed knowledge of how media excitations are coupled to electromagnetic waves. In electrically conducting media, an accurate description of propagating electromagnetic waves is a non-trivial problem because of non-local light-matter interactions. Among other consequences, the non-locality gives rise to the anomalous (ASE) and superanomalous (SASE) skin effects. As is well known, ASE is related to an increase in the electromagnetic field absorption in the radio frequency domain. This work demonstrates that the Landau damping underlying SASE gives rise to another absorption peak at optical frequencies. In contrast to ASE, SASE suppresses only the longitudinal field component, and this difference results in the strong polarization dependence of the absorption. The mechanism behind the suppression is generic and is observed also in plasma. Neither SASE, nor the corresponding light absorption increase can be described using popular simplified models for the non-local dielectric response.

## Introduction

Propagation and scattering of electromagnetic (EM) wave in conductive media, e.g. in metals, is a physical problem of fundamental importance. Delocalized charged carriers in such media screen the EM field modifying both reflection and absorption. The screening is referred to as *skin effect*^1,2^. Two types of the skin effect are commonly distinguished: the normal (NSE) one with the exponential spatial decay of the field, and the anomalous (ASE) skin effect with the power law decay. The field profile inside the media is not directly accessible in experiment, but the skin effect determines the penetration interval, where the field interacts with the media and loses its energy. This changes the surface impedance and, thus, the light scattering.

In the Hagen-Rubens regime of lower frequencies $\omega \ll 1/\tau$ ($\tau$ is the decay time), the dielectric response is essentially local, and the permittivity is well approximated by the Drude expression^3–5^. This leads to the normal skin effect with the exponential field decay $E\propto \exp (-z/a)$, where *z* is the distance from the metal surface, and *a* is the characteristic decay length^6,7^. In this regime, the frequency dependence of the surface impedance follows the law $\propto \omega ^{1/2}$.

At higher frequencies of $\omega \tau \gtrsim 1$, which corresponds to the microwave range for normal metals, the dielectric response is non-local, and one observes the ASE with the power law field decay $E\propto 1/z^{3}$^8–12^. The frequency dependence for the surface impedance becomes $\propto \omega ^{2/3}$. The increasing field penetration length in the ASE regime enhances the field losses leading to a larger absorption of the scattered light, manifested as a peak in the logarithmic frequency spectrum^8,9,11,12^.

When the frequency increases further, to the optical range in normal metals, the absorption demonstrates another peaked enhancement^13^, which is usually connected to the ASE as well^14^. However, in the interval of frequencies and wave vectors that corresponds to the scattered light, the dielectric response of the metal is practically local, and the skin effect is normal^6^. Also, unlike the absorption increase at lower frequencies, the higher frequency second peak depends strongly on the field polarization and on the scattering angle. It disappears for the normal angle scattering.

It has been recently shown that in the optical frequency range a metal can demonstrate the so-called superanomalous skin effect (SASE), where the field decays as $E\propto 1/(z\ln z^{2})$^15,16^. However, the SASE takes place only for large wave vectors $q \sim \omega /v_F$, where $v_F$ is the Fermi velocity of metallic electrons. This interval of *q* and $\omega$ corresponds to surface plasmons. It is thus unclear how the SASE can influence the scattering of light with much smaller wave vectors, $q \sim \omega /c$ ($c \gg v_F$, *c* is the speed of light).

In this work we revisit the classical problem of scattering of EM waves on a metallic surface using an exact solution of the EM problem with the non-local Lindhard-Reuter-Sondheimer permittivity. We demonstrate that despite the mismatch of the wave vectors, the second absorption peak is directly connected to the SASE not ASE. It is shown that the two absorption peaks are facilitated by different loss mechanisms that depend on the field polarization. While the ASE losses affect the transverse field component, the SASE suppresses the longitudinal one, similarly to the conventional Landau damping. This explains the polarization dependence of the second absorption peak and the fact it disappears for the normal scattering.

It is also shown that popular approximations for the non-local dielectric response, such as hydrodynamic and single-pole models^17–22^, fail to capture the SASE absorption peak and one needs more advanced models that take into account kinetic properties of metallic electrons^8,13,22,23^. This fact is very important for modelling optical properties of conductive media when the non-locality in the dielectric response plays a significant role^24^, e.g. to study propagation of plasmons in nanostructures^25–28^, near-field radiative heat transfer^29^, thermal and zero-point EM energy and forces on curved metallic boundaries^30^, losses in EM emitters placed near a metallic surface^31^, and propagation of EM waves in plasma^14^.

The results are presented as follows. We first outline the solution for the light scattering problem for a metallic surface with an arbitrary dielectric response of the metal. Then, using this solution, we calculate the absorptivity frequency spectrum, introduce the SASE and ASE and discuss their relation to the scattering characteristics. We demonstrate that a popular single-pole approximation fails to describe the SASE and its contribution to the absorption. A generic nature of the SASE- as well as ASE-related loss mechanisms is illustrated by considering a classical Maxwellian plasma. Finally, we summarize the results and discuss their significance.

**Figure 1:**
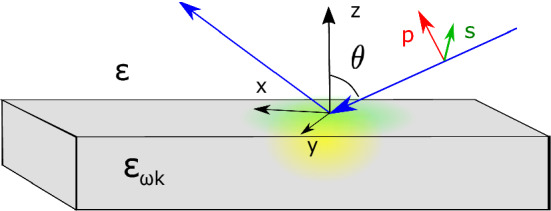
Illustration of the problem geometry. A wave approaching at the incident angle $\theta$ is reflected from a surface of a metal at $z=0$. TM (*p*) and TE (*s*) polarizations differ by the orientation of the electric field with respect to the surface, the field penetrating the sample is schematically illustrated by green and yellow, representing ASE and SASE contributions.

## Theory

### Scattering problem and its solution

The absorption spectrum is obtained by solving the scattering problem for an EM wave reflected from a metal-dielectric interface as shown in Fig. 1, where the interface at $z=0$ separates the metal at $z<0$ from the insulating media at $z>0$. The wave approaches the surface from the half-space $z > 0$ at incident angle $\theta$ and has the wave vector $\textbf{k}^{\textrm{in}} = k^{\textrm{in}} (\sin (\theta ), 0, - \cos (\theta ))$ with $k^{\text {in}} = \sqrt{\varepsilon _{h}}\, \omega /c$, and $\varepsilon _{h}$ is the dielectric constant of the insulating half-space, assumed $\varepsilon _{h} =1$ (vacuum) for simplicity. The scattering problem is solved by matching solutions of the Maxwell equations at the metal-insulator interface assuming the total solution is a mixture of the incoming and reflected waves at infinity $z \rightarrow +\infty$^1^.

The dielectric response of the metal will be taken into account by using results for the kinetic model that describes metal electrons. Further, we assume the metal-vacuum interface is infinitely thin. Thereby, we neglect the finite-width effects such as depletion of the electron density in the vicinity of the interface. We also consider a specular reflection model (SRM) for metal electrons scattering at the interface )diffusion parameter^13,32^ $p=1$), which is widely employed in studying surface related effects^6,8,10,18,19,21,33^. A model where weakly interacting metallic electrons are described by the Boltzman equation with the SRM for the scattering at the interface^8,13^ is also referred to as the semiclassical model (SCM) in the literature^22,23^ Although the adopted model appears simplified, it captures essential physics of the the SASE for as long as the characteristic skin length much exceeds the actual width of the interface. In normal metals the microscopic width of the interface is estimated as the inverse of the Fermi wave number $a \simeq 1/k_F \lesssim 1$nm^34^, which much smaller than other pertinent characteristic lengths in the problem, including the skin width, estimated as $l\gtrsim 10$ nm. We also argue that surface roughness and the violation of the SRM should not change our main conclusions.

The adopted model for the electrons and the interface means the normal component of the electrical current at the interface vanishes, which implies a certain boundary condition for the respective polarization component $\dot{\textbf{P}}_\perp =0$ [see Supplemental Material]. The scattering problem can thus be solved using an approach in the spirit of the ABC method^35^, where the Maxwell equations are complemented with the additional boundary condition [see Supplemental Material]. The solution to the Maxwell equations is obtained in a general form once the bulk dielectric response (permittivity tensor) of the metal is known (the magnetic response can be neglected in this case^36^). The consistency of this approach requires the boundary conditions for the polarization correspond to the kinetic model for metallic electrons. It is ensured for the SCM model, for which one obtains an exact solution of the EM problem. However, in many other situations, in particular, for semiconductors with excitons, the validity of the ABC is debated^22,23,37–41^.

Solving Maxwell equations on each side of the interface and matching the solutions at it is relatively straightforward albeit tedious. An interested reader can find details of the calculations in the Supplemental Material. The problem admits two independent solutions that differ by the field polarization relative to the interface^42^. For TM (*p*) polarized waves, the magnetic field is parallel to the interface, while for TE (*s*) polarized waves, the electric field is parallel. The reflection coefficient for both cases is given as

- *s*-wave polazrization:
  1a $$\begin{aligned} R_{s}&= \frac{Z_{s} - 1}{Z_{s} + 1}, \end{aligned}$$
  1b $$\begin{aligned}{}&Z_s = \frac{k_{z}^{\text {in}}}{2\pi } \int _{-\infty }^{\infty } \frac{ \text {d} k_z}{2\pi } G_{\omega {\textbf{k}}}, \end{aligned}$$
- *p*-wave polarization:
  1c $$\begin{aligned} R_{p}&= \frac{1-Z_{p} }{1+Z_{p}}, \end{aligned}$$
  1d $$\begin{aligned}{}&Z_{ p } = - \frac{1}{2 \pi k^{\textrm{in}}_{z}} \int _{-\infty }^{\infty } \frac{ \text {d}k_z}{2\pi } \frac{ G_{\omega {\textbf{k}}} }{\varepsilon ^{\ell }_{\omega \textbf{k}}} T_{\omega \textbf{k}}, \end{aligned}$$
  where
  1e $$\begin{aligned}{}&T_{\omega \textbf{k}} = q^{2} -{\bar{\omega }}^{2} \varepsilon ^{\ell }_{\omega \textbf{k}} +\frac{q^{2} {\bar{\omega }}^{2}}{k^{2}} \left( \varepsilon ^{\ell }_{\omega \textbf{k}} - \varepsilon ^{\text {tr}}_{\omega \textbf{k}} \right) , \end{aligned}$$
  1f $$\begin{aligned}{}&G_{\omega {\textbf{k}}}=\frac{-4\pi \,\mathbbm {i}}{k^{2}- \varepsilon _{\omega {\textbf{k}}}^{\textrm{tr}} \,\bar{\omega }^{2}}. \end{aligned}$$

Here we use notations ${\bar{\omega }} = \omega /c$, $k=\sqrt{q^{2}+k_{z}^{2}}$, $\textbf{q} = (q,0,0)$ is an in-plane wavevector that, without loss of generality, is assumed to have *x*-component only, and other quantities are related to the incoming wave as $k_{z}^{\text {in}} = k^{\text {in}}\,\cos (\theta )$, $q=k^{\text {in}}\,\sin (\theta )$, $k^{\text {in}} = \sqrt{q^{2}+ k_{z}^{\text {in}\,2}}$. Finally, the transverse $\varepsilon _{\omega \textbf{k}}^{\text {tr}}$ and longitudinal $\varepsilon _{\omega \textbf{k}}^{\ell }$ components define the permittivity tensor of the bulk metal. We note, that the contribution proportional to the difference $\varepsilon ^{\ell }_{\omega \textbf{k}} - \varepsilon ^{\text {tr}}_{\omega \textbf{k}}$ in Eq. (1e) is negligible in the non-relativistic limit where the average velocity of the carriers is much smaller than the velocity of light ($v_F \ll c$). In what follows, we will neglect this contribution.

### Dielectric permittivity of a metal

The dielectric response of a normal metal is a sum of ion polarization and a contribution of freely moving charge carriers (electrons). The latter comprises the part due to interband electronic transitions and the part induced by electrons moving within the same conduction band - intraband scattering. In this work, we are interested in the latter because it yields the non-local dielectric response. It is well described using the model of weakly interacting particles with a quadratic energy dispersion and a spherical Fermi surface. This model gives a permittivity tensor with the longitudinal (Lindhard)^18–21^ and transverse (Reuter & Sondheimer)^8,10^ components given as

2a $$\begin{aligned}{}&\varepsilon _{\omega \textbf{k}}^{\textrm{tr}} = 1 - \frac{3}{2k^2} \frac{\Omega }{\omega } \left[ 1 - \frac{1}{2} \left( \frac{\Omega }{k} - \frac{k}{\Omega } \right) \ln \left( \frac{\Omega - k}{\Omega + k} \right) \right] , \end{aligned}$$

2b $$\begin{aligned}{}&\varepsilon _{\omega \textbf{k}}^{\ell } = 1 + \frac{3}{k^2} \frac{\Omega }{\omega } \left[ 1 - \frac{\Omega }{2k} \ln \left( \frac{\Omega - k}{\Omega + k} \right) \right] , \end{aligned}$$

which will be referred to as the Lindhard-Reuter-Sondheimer (LRS) model. The expressions are written using scaled quantities $\Omega \rightarrow \Omega /\Omega _p$, $\textbf{k} \rightarrow \textbf{k} v_F /\Omega _p$, $\Omega _p$ the plasma frequency, and $\Omega = \omega + \mathbbm {i} \gamma$. The temperature dependent decay rate $\gamma = 1/\tau$ takes into account all electronic relaxation processes, e.g., electron-electron and electron-phonon scattering. Note, that accounting of the relaxation in Eqs. (2) follows from the perturbation theory of the kinetic equation describing charge carriers in metals as prescribed in Ref.^43^, and does not reduce to a simple substitution $\omega \rightarrow \omega + \mathbbm {i} \gamma$.

The LRS model contains three material parameters, $\Omega _p$, $v_F$, and $\tau$, that determine the dielectric response. Strictly speaking, Eq. (2) are valid only when *k* is not too large, and need to be changed to the full expressions when *k* becomes comparable to the Fermi wave number $k_F$. However, those large *k* corrections introduce only relatively small quantitative changes (less than  20%) to the results and do not modify the conclusions.

The non-locality of the dielectric response is manifested by the $\textbf{k}$-dependence of $\varepsilon _{\omega {\textbf{k}}}^{\mathrm{tr,\ell }}$. In a bulk sample, this gives rise to that the permittivity is a function of the coordinate difference $\textbf{r} - \textbf{r}^\prime$, where $\textbf{r}$ is the position at which the polarization is measured, and $\textbf{r}^\prime$ is where the electric field is applied. In the limit $k\rightarrow 0$ both components of the permettivity tensor reduces to the same Drude expression

3 $$\begin{aligned} \varepsilon _\omega ^{D} = 1 - \frac{1}{\Omega \, \omega }, \end{aligned}$$

which neglects the non-locality.

In our numerical calculations below, we consider silver as the prototype metal with the following parameters (taken at $T\simeq 4~K$): $\hbar \Omega _{p}=8.6~eV$, $\gamma =0.026~eV$ and $v_{F}=1.39\times 10^{6}~m/sec$^44,45^.

## Absorption and skin effect

**Figure 2:**
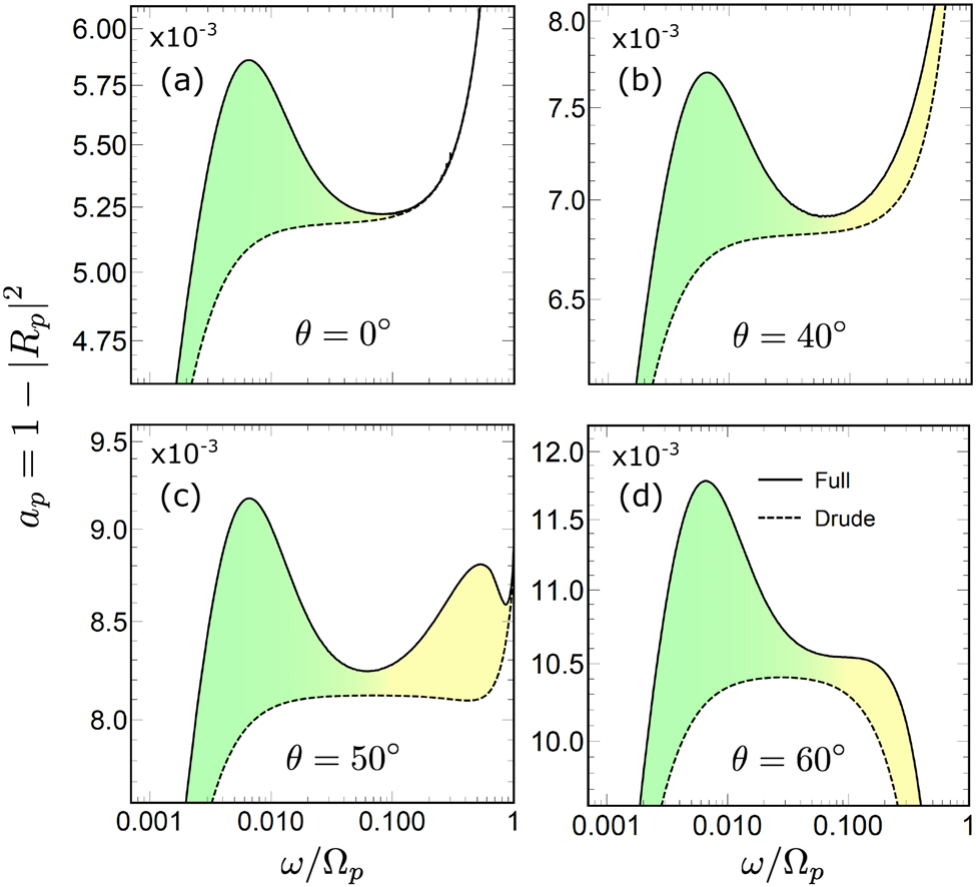
Frequency dependence of the absorptivity $a_p$ for *p*-polarized waves, calculated for incident angle values of $\theta = 0^\circ$ (**a**), $40^\circ$ (**b**), $50^\circ$ (**c**), and $60^\circ$ (**d**). Results for the LRS model in Eq. (4) are given by the solid like ”Full”, the Drude approximation is shown by the dashed line ”Drude”. Green/yellow colour filling represents the ASE/SASE frequency ranges.

### Absorptivity spectrum

The squared absolute value of the reflection coefficient is smaller than unity due to field absorption inside the metal. To quantify the loss we introduce the polarization dependent absorptivity as

4 $$\begin{aligned} a_{s,p} = 1- |R_{s,p}|^2. \end{aligned}$$

Figure 2 plots the frequency dependence (spectrum) of this quantity in the logarithmic frequency scale, calculated for *p*-polarized waves for a few incident angles $\theta$. Solid lines ”Full” are obtained using the complete LRS model in Eq. (2), while the dashed ”Drude” lines are calculated using the local Drude approximation in Eq. (3) for both permittivity components. The difference between the two is accentuated by the color filing where the green and yellow colours are used to mark, respectively, the frequency intervals of the ASE and SASE.

For the normal angle $\theta = 0^\circ$ scattering Fig. 2a reproduces a seminal work of Reuter and Sondheimer^8^. The difference between the Drude approximation and the full LRS model has a pronounced peak in the interval $10^{-3} \lesssim \omega \lesssim 10^{-1}$ with the maximum at $\omega \simeq 10^{-2}$. This peak is closely related to the ASE, and for silver, it takes place in the domain of microwave frequencies. Clearly, the absorptivity for *p* and *s* polarizations are the same at $\theta = 0^\circ$.

For other reflection angles, the difference between the LRS model and the Drude approximation appears at higher frequencies $\omega \gtrsim 0.1$ as well [Fig. 2b–d], but only for *p*-polarized waves. The difference is observed for all angles, but is most notable at $\theta \simeq 50^\circ$ where one sees is a second peak with the maximum at $\omega \simeq 0.4$ [Fig. 2c]. At small and large angles the peak is absent although the difference is still visible [Fig. 2b and d].

**Figure 3:**
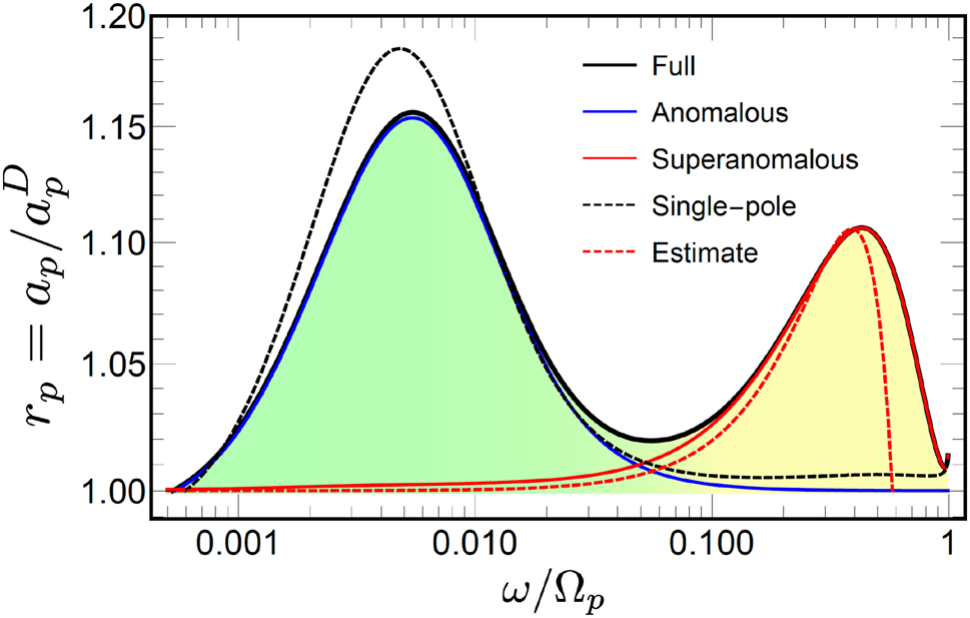
Relative absorptivity $r_p = a_p/a^D_p$, where $a_p^D$ is obtained for the Drude model. The black line ”Full” gives $a_p$, calculated for the full model in Eqs. (2), the solid blue line ”Anomalous” and the red line ”Superanomalous” give $a_p$ obtained using A and S approximations in Eqs. (9) and (10), respectively. The dotted black line ”Single-pole” gives $a_p$ obtained using the single-pole approximation in Eq. (11). Red dashed line ”Estimate” give the estimation for $a_p$ given by Eq. (17). The colour filling corresponds to the ASE and SASE frequency ranges. The incident angle is $\theta = 50^\circ$ .

To highlight the non-locality role we consider the relative absorptivity

5 $$\begin{aligned} r_p = \frac{a_p}{a_p^D} = \frac{1- |R_p|^2}{1- |R_p^D|^2}, \end{aligned}$$

where absorptivity $a_p$ and reflection coefficient $R_p$ are calculated for the full LRS model in Eq. (2), while $a_p^D$ and $R_p^D$ are obtained using Drude approximation. A black solid line marked ”Full” in Fig. 3 demonstrates the logarithmic frequency dependence of $r_p$ calculated at $\theta = 50^\circ$.

**Figure 4:**
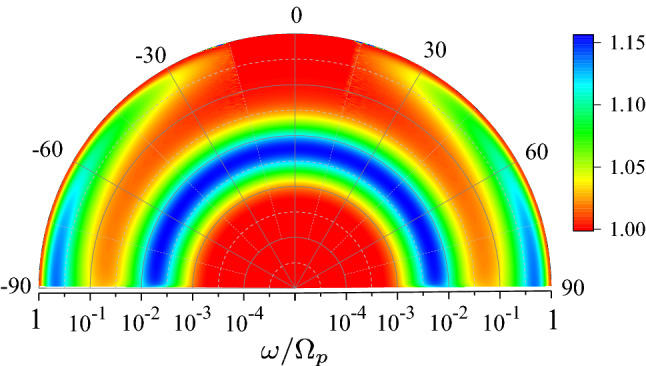
Colour-density plot of the relative absorptivity $r_p$ as a function of frequency $\omega$ and incident angle $\theta$ for $\alpha = 5\times 10^{-3}$.

The result demonstrates two clearly visible peaks of comparable amplitude, with the maxima at $\omega \simeq 0.005$ and $\omega \simeq 0.4$. The two-peak structure of the spectrum holds in a large interval of the incident angles. It is also illustrated by a colour-density Fig. 4, which plots $r_p$ as a function of $\omega$ and $\theta$. It shows clearly that the lower frequency peak is practically independent of the angle, whereas the higher frequency one decreases at smaller $\theta$ disappearing at $\theta \rightarrow 0$.

Connections between the first absorption peak at lower frequencies and the ASE was first noted in the original work of Reuter and Sondheimer^8^. The second higher frequency peak observed in later studies^13^ was similarly attributed to the ASE^14^. However, this explanation contradicts to the fact in metals the ASE is restricted to the interval $\omega \lesssim 0.05$, whereas at $\omega \gtrsim 0.05$ and $q \simeq \omega /c$ the permittivity is well approximated by the local Drude expression, and the skin effect is normal. In principle, the frequency interval $\omega \gtrsim 0.1$ of the second peak corresponds to the SASE^15^. However, the latter takes place at wave vectors $q \simeq \omega$ much larger than those for the scattered light $q_L \le \alpha \omega$, $\alpha = v_F/ c \ll 1$.

### SASE vs ASE

We now briefly discuss the origin of SASE and ASE and differences between them. The electric field parallel to the interface in the Fourier $\textbf{k}$-space is found as (see Supplemental Material)

6 $$\begin{aligned} E_x \propto \frac{ G_{\omega {\textbf{k}}} }{\varepsilon _{\omega {\textbf{k}}}^{\ell }} T_{\omega {\textbf{k}}}, \end{aligned}$$

where one needs to substitute ${\bar{\omega }} = \alpha \omega$. To obtain spatial decay of the field inside the metal one needs to calculate the inverse Fourier transform of the solution over $k_z$. The type of the skin effect is defined by the field asymptotic at large *z*, which is determined by singularities of Eq. (6) in the $\textbf{k}$-space^15^. At the same time, the surface impedance in Eq. (1d) is given by the field amplitude at the interface $z=0$, calculated by integrating Eq. (6) over $k_z$.

Equation (6) can be split using the following identity

7 $$\begin{aligned}{}&\frac{G_{\omega {\textbf{k}}}}{\varepsilon ^{\ell }_{\omega {\textbf{k}}}} = \frac{1}{N_{\omega {\textbf{k}}}} \left\{ \frac{-4\pi \mathbbm {i}}{\varepsilon ^{\ell }_{\omega {\textbf{k}}} } - \frac{\alpha ^2 \omega ^2}{2} \left( 1 - \frac{k^2}{\Omega ^2} \right) G_{\omega {\textbf{k}}} \right\} , \end{aligned}$$

where

$$\begin{aligned}{}&N_{\omega {\textbf{k}}} = \frac{3 \alpha ^2}{2} \frac{\omega }{\Omega } (1 - \Omega \omega ) + k^2 \left( 1 + \frac{\alpha ^2}{2} \frac{\omega ^2}{\Omega ^2} \right) . \end{aligned}$$

It is possible to demonstrate that the longitudinal and transverse contributions to the field inside the metal are proportional, respectively, to $1/\varepsilon _{\omega \textbf{k}}^{\ell }$ and $G_{\omega \textbf{k}}$ in Eq. (7), where the latter depends on $\varepsilon _{\omega \textbf{k}}^{\textrm{tr}}$ only.

In the Hagen-Rubens regime of low frequencies, both permittivity components are approximated by the Drude expression, which gives an exponential NSE asymptotic for the field. At larger frequencies, the Drude approximation breaks down, and Eqs. (2) must be used. The ASE asymptotic $E \propto 1/z^3$ is obtained from the transverse field component, which depends on $\varepsilon _{\omega \textbf{k}}^{\textrm{tr}}$ only. In contrast, the SASE asymptotic $E \propto 1/z \ln z^2$ is given by the longitudinal field component that depends on $\varepsilon _{\omega \textbf{k}}^{\ell }$^15^.

From the mathematical perspective, the difference between the ASE and SASE appears due to different singularity types in the transverse and longitudinal field components. To demonstrate this we note that the ASE is observed at small frequencies $\omega \ll \alpha$, where one can expand Eq. (2a) assuming also $\omega \ll k$. Keeping the largest contributions containing $\omega$ to the Green function and neglecting $\gamma$ one obtains

8 $$\begin{aligned} G_{\omega {\textbf{k}}} \simeq \frac{- \mathbbm {i} 4 \pi }{ k^2 - \mathbbm {i} \pi 3 \alpha ^2 \omega / 4 k}, \end{aligned}$$

a well known approximation in the theory of ASE^6^. The imaginary part of the *k*-dependent denominator describes the losses, appearing physically due to the energy transfer to a bath of collisionless electrons. This aspect of the ASE loss mechanism is similar to the Landau damping, and is sometimes referred to as such^6,46,47^. However, in contrast to the standard Landau damping, here the energy is transferred to electrons moving perpendicular to the vector $\textbf{k}$ of the wave propagation. Notice also, that the imaginary part in Eq. (8) is small when $\omega /k \ll 1$.

As a simple example, we consider the normal scattering at $\theta =0$. The Green function in Eq. (8) has a pole at $k_z = (i \pi 3 \alpha ^2 \omega /4)^{1/3}$. The inverse Fourier integral over $k_z$ yields the well known ASE frequency asymptotic $\delta \propto \alpha ^{-2/3} \omega ^{-1/3}$ for the skin depth $\delta$^6^. However, when the frequency increases to $\omega \gtrsim \alpha$, the non-locality becomes negligible, and one can use the Drude expression for the permittivity, which gives the NSE.

In contrast, the SASE contribution, which is proportional to $1/\varepsilon ^{\ell }_{\omega {\textbf{k}}}$ in Eq. (7), does not have poles in the vicinity of real $k_z$. This can be easily seen from the fact that the equation for the poles $\varepsilon ^{\ell }_{\omega {\textbf{k}}} =0$ gives the frequency dispersion of bulk plasmons, which has (almost) real solutions only if $\omega > \Omega _p$. A non-zero imaginary part of $\varepsilon ^{\ell }_{\omega {\textbf{k}}}$, appearing at at $\omega < k$, is a manifestation of the Landau damping acting on the longitudinal field component.

The logarithmic branch-cut of $\varepsilon ^{\ell }_{\omega {\textbf{k}}}$ in the LRS model (2) yields the largest contribution to the integral over $k_z$ of the inverse Fourier transform of Eq. (6). It gives a non-exponential SASE field asymptotic but only if the in-plane wave vector is not small, i.e. at $q \simeq \omega$^15^. In the limit of small *q* the dielectric response is well described by the Drude model, and the field decays exponentially at large *z*^6^. Notice, the *s*-polarized solution contains only $G_{\omega \textbf{k}}$, and thus does not exhibit the SASE.

### ASE and SASE contribution to absorption

The relation between the transverse and longitudinal permittivity components on one side and the ASE and SASE on the other provides one a simple tool to separate the respective contributions to the absorptivity. We make use of that the SASE is determined by the longitudinal component of the permittivity tensor only. This follows from a possibility to separate the solution for the electric field as shown in Eq. (7) into the parts depending on $\varepsilon _{\omega {\textbf{k}}}^{\textrm{tr}}$ and $\varepsilon _{\omega {\textbf{k}}}^{\ell }$. The latter contribution gives rise to the SASE long range asymptotic for the electric field inside the metal, whereas the former yields the standard ASE asymptotic^15^. Using the Drude expression for $\varepsilon _{\omega {\textbf{k}}}^{\textrm{tr}}$ or $\varepsilon _{\omega {\textbf{k}}}^{\ell }$ eliminates the ASE and SASE power law asymptotic, correspondingly.

We use this observation and introduce two approximations we call A (”Anomalous”) and S (”Superanomalous”). In A approximation, we assume $\varepsilon _{\omega {\textbf{k}}}^{\textrm{tr}}$ is given by the LRS model (2a), and for $\varepsilon _{\omega {\textbf{k}}}^{\ell }$ we use the Drude expression (3). The surface impedance in this case reads as

9 $$\begin{aligned} Z_p^{A} =&\frac{ \mathbbm {i} }{k^{\textrm{in}}_z} \int _{-\infty }^{\infty } \frac{\textrm{d} k_z}{\pi } \frac{ q^2/\varepsilon _{\omega }^{D} - {\bar{\omega }}^2 }{k^{2} - \varepsilon _{\omega {\textbf{k}}}^{\textrm{tr}} {\bar{\omega }}^{2}}. \end{aligned}$$

This approximation keeps the ASE contribution to the field, but eliminates the SASE one.

In S approximation, we take, respectively, the LRS model (2b) for $\varepsilon _{\omega {\textbf{k}}}^{\ell }$, and the Drude approximation for $\varepsilon _{\omega {\textbf{k}}}^{\textrm{tr}}$. This yields the impedance as

10 $$\begin{aligned} Z_p^{S} =&\frac{ \mathbbm {i} }{k^{\textrm{in}}_z} \int _{-\infty }^{\infty } \frac{\textrm{d} k_z}{\pi } \frac{ q^2/ \varepsilon _{\omega {\textbf{k}}}^{\ell } - {\bar{\omega }}^2 }{k^{2} - \varepsilon _\omega ^{D} {\bar{\omega }}^{2}}. \end{aligned}$$

This approximation keeps the SASE contribution to the field, and the ASE one is omitted.

We now use A and S approximations are to calculate the relative absorptivity $r_p$. The results are shown in Fig. 3 by blue and red lines, respectively. Approximation A excellently reproduces the lower frequency peak, but fails to capture the higher frequency one completely. In contrast, approximation S captures the higher frequency peak but misses the lower frequency one completely. This establishes a direct relation between the absorptivity peaks on one side, and the ASE and SASE on the other.

### Single-pole approximation

Qualitative differences between the two peaks can be also clearly demonstrated by adopting a popular Silin-Klimontovich-Lindhard model for the permittivity^18–21^. This model takes a non-local nature of the metal dielectric response into account by amending the Drude expression with an additional $\textbf{k}$-dependence of its pole position, which is also referred to as the single-pole approximation. Formally, this approximation can be derived from Eqs. (2) by applying the perturbation expansion to the permittivity tensor at small $k\ll \omega$, where only the leading order corrections are kept. Collecting all *k*-dependent terms into the denominator one obtains the transverse permittivity as

11 $$\begin{aligned}{}&\varepsilon _{\omega \textbf{k}}^{\mathrm{tr,\ell }} = 1- \frac{1}{\omega \Omega - c_\mathrm{tr,\ell } k^2 }, \end{aligned}$$

where $c_{\textrm{tr}} = \omega /5\Omega$ and $c_{\ell } = 3 \omega / 5\Omega$. It is, however, restricted only to small *k*’s. This expression can be also obtained using a popular hydrodynamic model for the dielectric response^22,34^, which, however, yields different coefficients $c_\mathrm{tr,\ell }=\omega /3\Omega$. In this case, the obtained the model has no limitation on values of *k*^48^ It is commonly assumed that the single-pole approximation describes all physically relevant effects due to non-locality in the dielectric response^22^. However, as we will demonstrate it is not the case with the SASE and the related absorption peak at higher frequencies.

The relative absorptivity $r_p$ calculated with the single-pole model (11) is shown in Fig. 3 by a dotted black line. The model qualitatively reproduces the first ASE peak but fails to capture the second one completely. This deficiency of the model is closely related to the origin of the SASE and deserves a closer look. As shown above, the ASE contribution to the impedance in Eq. (1d) is defined by singularities of $G_{\omega \textbf{k}}$. Using the single-pole approximation in Eq. (11) and taking the limit $\omega \ll \gamma$, one obtains the following approximate expression

12 $$\begin{aligned} G_{\omega {\textbf{k}}} \simeq \frac{- \mathbbm {i} 4 \pi }{ k^2 - \mathbbm {i} 5 \alpha ^2 \gamma \omega / k^2}. \end{aligned}$$

Comparing this expression with Eq. (8) obtained from the original LRS model (2) one sees that it also has a pole singularity in the vicinity of the real $k_z$ axis. Using this approximation one obtains the frequency asymptotic of the surface impedance as $Z_p \propto \omega ^{3/4}$, which overestimates the result $Z_p \propto \omega ^{2/3}$ obtained from Eq. (8). Nevetherless, the single-pole approximation yields the maximum of the absorptivity peak at $\omega \simeq \alpha$, which is close to the result obtained with the LRS model [cf. Fig. 3].

In contrast, the SASE contribution to the impedance ($\propto 1/\varepsilon _{\textbf{k},\omega }^{\ell }$) has a different singularity type. It does not have a pole close to the real $k_z$ axis, and the main part of the corresponding integral for the impedance comes from the logarithmic branch-cut. The single-pole approximation does not reproduce this type of singularity and, as a consequence, fails to capture the SASE peak. We note in passing that the result cannot be improved by using many-pole extensions of the model.

**Figure 5:**
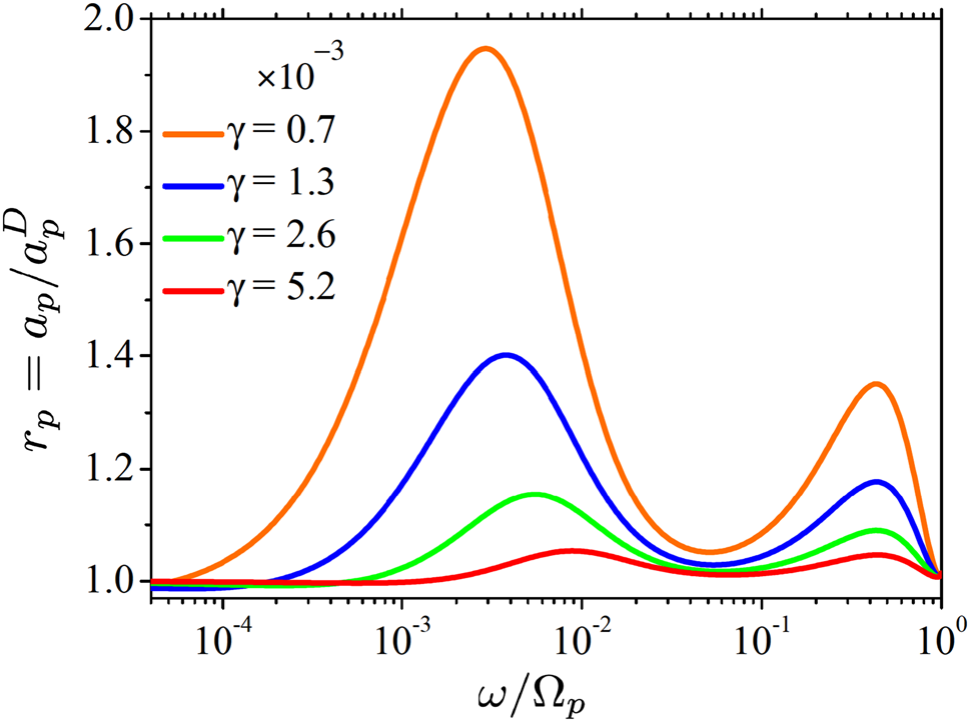
Relative absorptivity $r_p$, calculated for selected values of the decay rate $\gamma$ (shown in units of $\Omega _p$) for incident angle $\theta = 60^\circ$.

### SASE-induced absorptivity peak

It is important to explain how the SASE, which takes place in the domain of plasmons $q \simeq \omega$^15^, affects scattering of light waves with much smaller $q \simeq \alpha \omega \ll \omega$. Notice, that there is no such problem for the ASE peak, because ASE takes place at very small *q* as well. An explanation of this inconsistency for SASE is that scattering of long EM waves on the interface generates plasmon modes of much shorter wavelength, which are damped more efficiently.

To demonstrate this, we estimate the SASE contribution to the surface impedance using S approximation in Eq. (10), which we split into two parts as

13 $$\begin{aligned}{}&Z_{p}^{S} = Z_p^{D} + \delta Z_p^{S}. \end{aligned}$$

Here $Z_p^{D}$ is the Drude contribution to the impedance, where Eq. (3) is used for both permittivity components, while correction $\delta Z_p^{S}$ writes as

14 $$\begin{aligned} \delta Z_p^{S} = \frac{ \mathbbm {i} q^2}{ k_z^{\textrm{in}} } \int _{-\infty }^{\infty } \frac{\textrm{d} k_z}{\pi } \frac{1/\varepsilon _{\omega {\textbf{k}}}^{\ell } -1/\varepsilon _\omega ^{D} }{k^{2} - \varepsilon _\omega ^{D} {\bar{\omega }}^{2}}. \end{aligned}$$

Taking into account that $\alpha \ll 1$ we consider the quasistatic approximation of $\alpha = 0$. We also recall the inequality $q \ll \omega$ for the reflected light wave and apply the series expansion with respect to *q* for the integrand, where we keep only the leading order contributions. This yields

15 $$\begin{aligned} \delta Z_p^{S} \simeq \frac{ 2 \mathbbm {i} q^2}{\pi k_z^{\textrm{in}} } \int _{0}^{\infty } \left( \frac{1}{ \varepsilon _{\omega k_z}^{\ell }} - \frac{1}{\varepsilon _\omega ^{D}} \right) \frac{\textrm{d} k_z}{k_z^2}. \end{aligned}$$

This integral is estimated using the steepest descent approach [see Supplemental Material]. Assuming the result is small one obtains the corresponding relative absorptivity as

16 $$\begin{aligned} r_p^S \simeq 1 + \frac{\textrm{Re}[\delta Z_p^{S}]}{\textrm{Re}[Z_p^{D}]}. \end{aligned}$$

The Drude part $Z_p^{D}$ is easily calculated analytically. Substituting the result of the saddle-point calculations for $\delta Z_p^{S}$ one obtains

17 $$\begin{aligned} r_p^S \simeq 1 + 1.2\, \frac{ \alpha }{\gamma } \sin ^2(\theta ) \, \omega ^{5/3} (1 - \omega ^2)^{3}. \end{aligned}$$

This approximation, represented by the red dashed line in Fig. 3, gives a reasonably good agreement with with both the LRS model and S approximation for the second peak.

**Figure 6:**
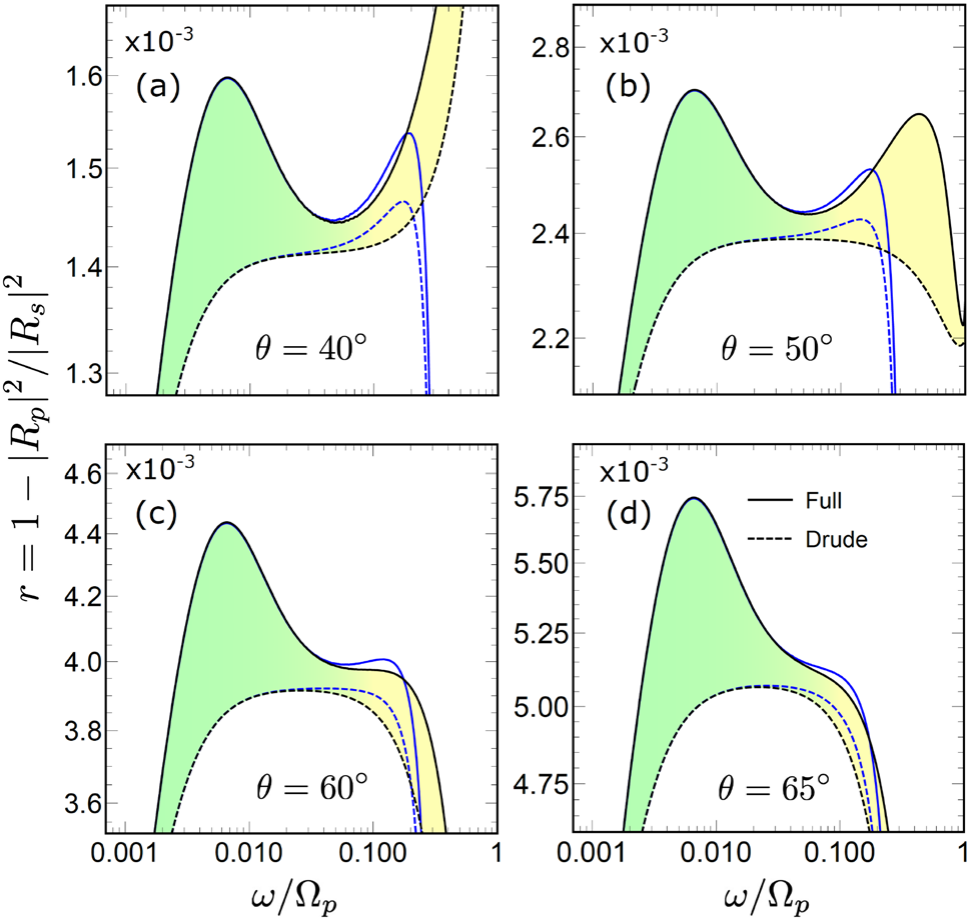
Reflectivities ratio $r=1- |R_p|^2/|R_s|^2$ subtracted from unity for *p* and *s* polarizations, for $\theta = 40^\circ$ (a), $\theta = 50^\circ$ (b), $\theta = 60^\circ$ (c) and $\theta = 65^\circ$ (d). Results for the full LRS model (solid line ”Full”) are compared with the Drude approximation (dashed line ”Drude”). Blue lines give the results calculated with the interband transitions, solid and dashed lines represent the full and Drude models, respectively.

According to Eq. (17), the maximal deviation from the Drude result depends on the ratio of two small constants, $\alpha /\gamma$, and can thus have an appreciable value. The result agrees with the intuitive expectation that the relative SASE contribution to the absorptivity increases at smaller values of $\gamma$. This is supported by numerical calculations for the full LRS model that are shown in Fig. 5, where both ASE and SASE relative contributions are calculated for several values of $\gamma$. We note in passing an interesting trend that the relative amplitude of the SASE peak grows at larger $\gamma$ compare to that of the ASE peak.

Details of the analytical calculations of the integral in Eq. (15) reveal the exact relation between the SASE and the second absorptivity peak. The absorptivity enhancement is determined by $\textrm{Re}[\delta Z_p^{S}]$, i.e. by the imaginary part of the integral in Eq. (15). The latter depends on $\textrm{Im}[\varepsilon _{\omega k_z}^{\ell }]$, which is non-zero at $k > \omega$, i.e. when the Landau damping sets in. The calculation shows that the main contribution to the integral in Eq. (15) is given by the interval $k_z \gtrsim \omega$, which is precisely the domain of surface plasmons where the SASE is observed^15^. To summarize the damping mechanism works as follows. When the light is reflected from a surface at a non-normal angle, a wide continuum of surface plasmon waves is generated. It includes plasmons with $k \gtrsim \omega$, for which the damping is significant due to SASE. However, it affects only the longitudinal field component and, therefore, the damping increase at larger reflection angles where the longitudinal component grows.

It is of interest to note that for the SASE contribution Eq. (17) yields the frequency asymptotic of $\omega ^{5/3}$, which differs from both $\omega ^{1/2}$ of the NSE and $\omega ^{2/3}$ of the ASE. This result, however, is to be taken with a grain of salt because for small $\omega$ the integral for the impedance contains an additional logarithmic contribution^16^. Nevertheless, in the SASE frequency interval $\omega \gtrsim 0.1$, the estimate fits the numerical result with a reasonable accuracy.

**Figure 7:**
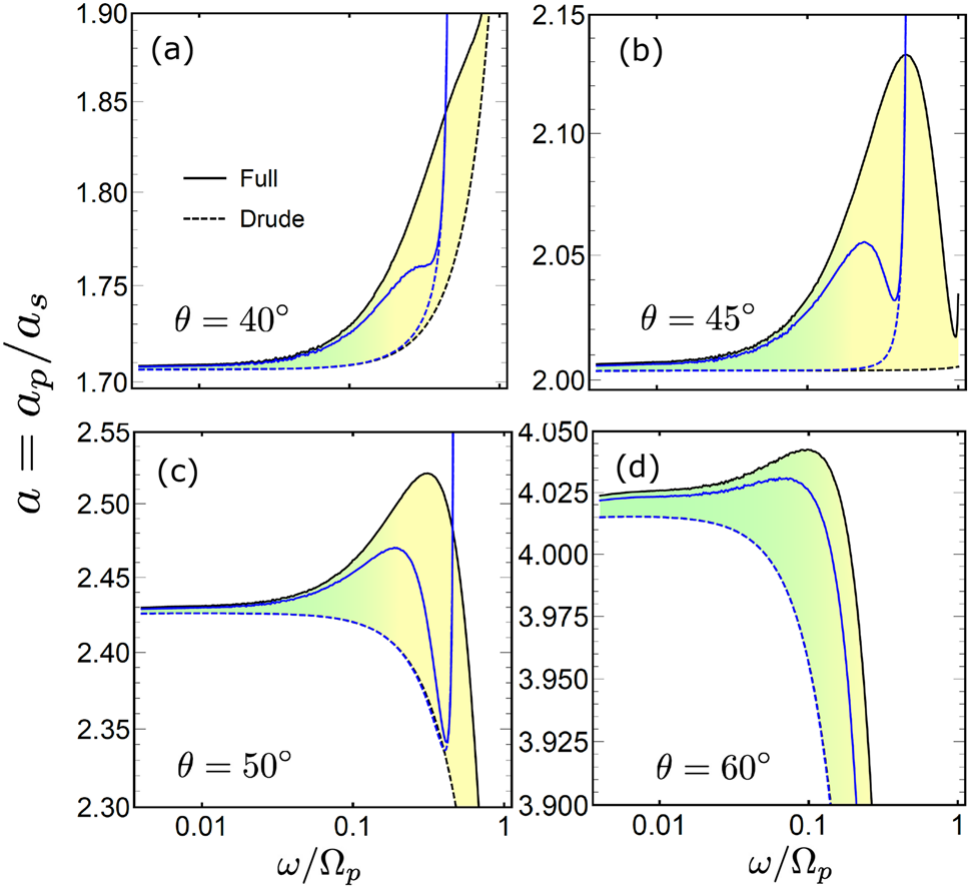
Absorptivities ratio $a=a_p/a_s$ for *p* and *s* polarizations, for $\theta = 40^\circ$ (**a**), $\theta = 45^\circ$ (**b**), $\theta = 50^\circ$ (**c**) and $\theta = 60^\circ$ (**d**). Results for the full model (solid line ”Full”) are compared with the Drude approximation (dashed line ”Drude”). Blue lines give the result with the interband transitions, solid and dashed lines represent the full and Drude models, respectively

### *s* and *p* polarizations

Form a practical point of view, it is interesting to consider two quantities where the SASE contribution can be seen most clearly. Note, that SASE affects only *p*-polarized waves, while for *s* polarization the effect is absent. To highlight this difference between the polarizations, one can consider the ratio of the *p* and *s* polarization reflectivities defined as^49^

18 $$\begin{aligned} r=1-\frac{|R_p|^2}{|R_s|^2}. \end{aligned}$$

This quantity is not sensitive to the radiation intensity, which is very advantageous in experiment.

The frequency dependence of *r* is shown in Fig. 6 for a few scattering angles $\theta$. Results for the full LRS model (2) are given by black solid lines, while dashed lines represent the Drude approximation. The results confirm the two-peak structure. The highest visibility of the second peak is observed at $\omega \simeq 50^\circ$, where both peaks have almost the same amplitude. At $\theta \lesssim 60^\circ$ the second peak gradually disappears even though the difference between the full and Drude models remains large [cf. Fig. 3]. Thus, appearance of the second peak in this quantity at higher frequencies, which changes with the scattering angle, is a clear manifestation of the SASE effect.

One can also consider another quantity where the influence of the SASE is seen even more directly

19 $$\begin{aligned} a = \frac{a_p}{a_s} = \frac{1- |R_p|^2}{1- |R_s|^2}. \end{aligned}$$

The frequency dependence of *a* calculated at different scattering angles is shown in Fig. 7. The dependence is practically constant in the ASE frequency domain, because the ASE enhances the absorptivity for both *s* and *p* polarizations similarly. However, this changes at higher frequencies, where *a* demonstrates a notable deviation from the constant. The SASE manifests itself via the appearance of the peak in the frequency dependence. The peak is absent when only the Drude contribution is taken into account, although it leads to the rise/decrease of *a* depending on the scattering angle [see Fig. 7].

### Visibility of the SASE peak

Finally, we comment on experimental observability of SASE peaks. In metals, the ASE and the corresponding peak takes place at relatively low frequencies where the influence of other factors on the absorption, like interband transitions, is minimal. This makes comparison of the theoretical predictions based of the LRS model with experimental measurements much easier^11,12^ In contrast, the SASE peak is found at much higher frequencies where the contribution of the interband transitions cannot be neglected. For the latter we adopt a standard model, where the LRS model (2) of the permittivity is supplemented with additional Lorentz contributions^50^ In the local limit of $k \rightarrow 0$ its yields the Drude-Lorentz model, frequently adopted to investigate plasmons^51^ For the calculations we use material parameters for silver^50,52,53^. An interested reader can find details of the model and calculations in the Supplemental Material. The results are shown in Figs. 6 and 7 by blue lines. They demonstrate that the SASE peak is still visible in both *r* and *a* quantities, although the transitions diminish its amplitude.

## Classical Maxwellian plasma

As demonstrated above, the additional absorptivity peak at higher frequencies is related to SASE. The mechanism underlying the absorptivity enhancement is the Landau damping of the longitudinal field, appearing due to surface plasmons generated by the light scattering on a surface. Mathematically, the losses are a consequence of the Fermi distribution of metallic electrons. However, both SASE and the related absorption peak are more general phenomena. The necessary requirement is the non-local dielectric response and collisionless Landau damping. We will show that SASE and the absorption peak are observed also in plasma as long as the surface plasmonic modes mix transverse and longitudinal field components^47^

**Figure 8:**
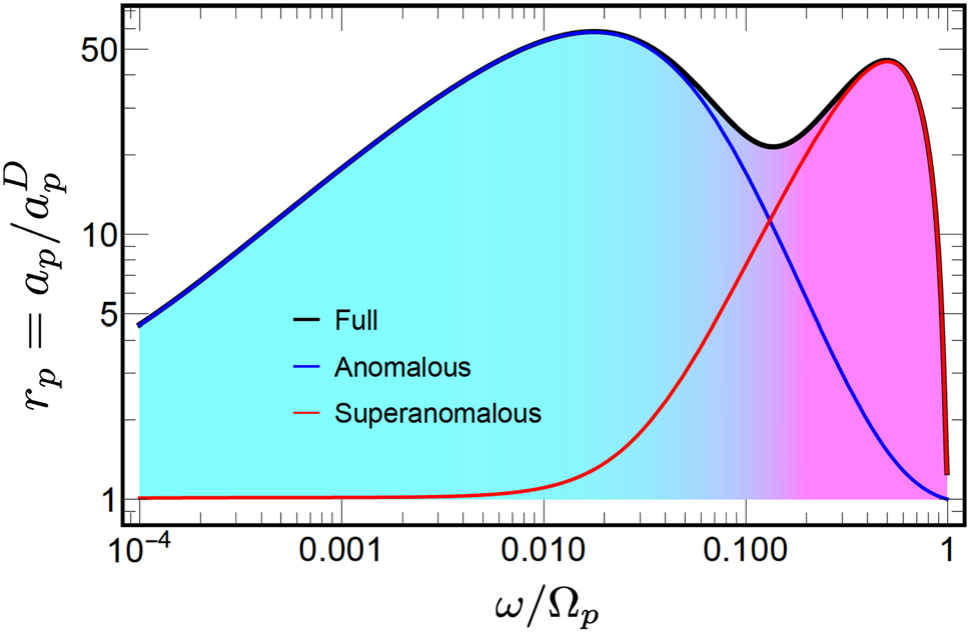
Relative absorptivity $r_p = a_p/a^D_p$ calculated for a Maxwell plasma, $a_p^D$ is the result of the Drude model. The black line ”Full” gives $a_p$ of the full model in Eqs. (20), the solid blue line ”Anomalous” and the red line ”Superanomalous” show $r_p$ obtained using the A and S approximations, defined by Eqs. (9) and (10), respectively. The cyan and magenta colour filling shows the frequency intervals of the ASE and SASE. The incident angle is $\theta = 50^\circ$.

We demonstrate it using a model of a classical non-relativistic plasma of electrons and ions with the Maxwell temperature distribution. Apart from the illustrative purposes, this analysis is useful for applications, because the problem of EM wave scattering of a plasma has practical aspects. For example, it has recently gained much attention in connection with the so-called plasma mirrors, designed to achieve high harmonic generation and even bring the Schwinger limit within reach^54^.

For the calculations, we assume a standard approximation where one neglects movements of heavier ions, which are regarded as stationary scattering centres introducing an additional decay rate $\gamma$ for rapidly moving electrons. The permittivity tensor for this model are given by its components^6,55^

20a $$\begin{aligned}{}&\varepsilon _{\omega \textbf{k}}^{\ell } = 1+ \frac{1}{(a k)^2} \frac{\Omega }{\omega } \left[ 1+ F \left( \frac{\Omega }{\sqrt{2} k v_T} \right) \right] , \end{aligned}$$

20b $$\begin{aligned}{}&\varepsilon _{\omega \textbf{k}}^{\textrm{tr}} = 1+ \frac{\Omega _e^2}{\Omega \omega } F \left( \frac{\Omega }{\sqrt{2} k v_T} \right) , \end{aligned}$$

where

21 $$\begin{aligned} F(x) = - \sqrt{\pi } x e^{-x^2} \left[ \mathbbm {i} + \textrm{Erfi} (x) \right] . \end{aligned}$$

is defined using imaginary error function $\textrm{Erfi}$^56,57^. The temperature velocity $v_T$ of electrons, the Debye radius *a*, and the electronic plasma frequency $\Omega _e$ are defined as

22 $$\begin{aligned} v_T = \sqrt{ \frac{T}{m_e}}, \quad a = \sqrt{\frac{T}{4\pi N_e e^2}}, \quad \Omega _e = \frac{v_T}{a}, \end{aligned}$$

where $N_{e}$ is the electronic density, $m_{e}$ is the electron mass, $\Omega =\omega +i\gamma$, and the loss constant for a fully ionized hydrogen plasma is estimated as^6,58–60^

23 $$\begin{aligned} \frac{\gamma }{\Omega _{e}}=\frac{2 \sqrt{2} e^3 \sqrt{N_e} \ln \left( a \sqrt{m T}/\hbar \right) }{3 T^{3/2}} \end{aligned}$$

As above, we introduced the scaled quantities $\omega \rightarrow \omega /\Omega _{e}$, ${\textbf{k}}\rightarrow {\textbf{k}} v_{T}/\Omega _{e}$, where $v_{T}$ and $\Omega _{e}$ plays the same role as $v_{F}$ and $\Omega _{p}$ in metals. For the calculations we assume parameters of a dense hot plasma accessible under laboratory conditions^60^ with the density $N_e=2 \times 10^{17} ~cm^{-3}$ and $T=2\times 10^{6}~K$. These parameters correspond to the temperature velocity $\alpha = v_{T}/c = 1.84\times 10^{-2}$ and the loss rate of $\gamma = 10^{-4}$. The effective plasma frequency for this density is $\Omega _{e} = 16.6~meV$.

**Figure 9:**
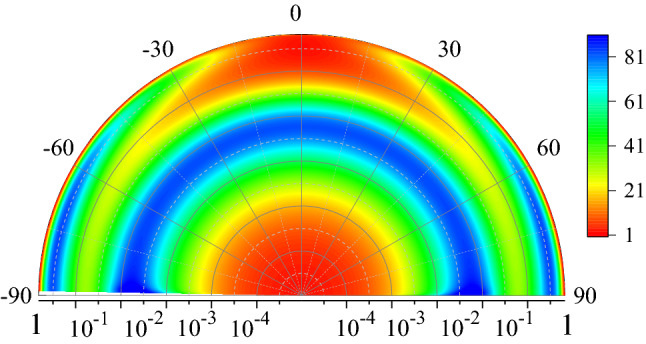
Colour-density plot for absorptivity ratio $r_p$ as a function of frequency $\omega$ and scattering angle $\theta$ in plasma for $\alpha = 1.84\times 10^{-2}$.

Figure 8 plots the relative absorptivity $r_p$ (5), calculated using the full model (20), and results of A and S approximations, defined by Eqs. (9) and (10), respectively. As in metals, $r_p$ has two peaks. The maximum of the lower frequency (ASE) peak is found at $\omega \simeq \alpha$, while the higher frequency (SASE) peak has its maximum at $\omega \simeq 0.5$.

As for metals, the ASE peak is determined by the transverse component of the permittivity tensor. For the corresponding frequency interval one obtains an approximate expression for the Green function similar to that in Eq. (8), but with different numerical coefficients. The SASE peak is related to the longitudinal field component and is determined by singularities in $1/\varepsilon _{\omega \textbf{k}}^{\ell }$. For plasma, the permittivity does not have logarithmic branching point and is complex for any (real) *k*. One sees this from the asymptotic expressions for the longitudinal permittivity that are

24a $$\begin{aligned}{}&\varepsilon _{\omega \textbf{k}}^\ell \simeq 1 - \mathbbm {i} \sqrt{\frac{\pi }{2}} \frac{\Delta ^2}{\omega k^3}\exp \left( - \frac{\omega ^2}{2k^2} \right) , \quad&k \ll \omega , \end{aligned}$$

24b $$\begin{aligned}{}&\varepsilon _{\omega \textbf{k}}^\ell \simeq 1+ \frac{1}{k^2} - \mathbbm {i} \sqrt{\frac{\pi }{2}} \frac{\omega }{k^3}, \quad&k \gg \omega . \end{aligned}$$

In contrast to the Lindhard expression (2b) for metals, here the small-*k* asymptotic is always complex. Its imaginary part is exponentially small at $k \rightarrow 0$, which implies the SASE contribution to the absorptivity vanishes in the limit $\omega \rightarrow 0$. However, the non-zero imaginary part increases the SASE contribution to the absorptivity at low frequencies, which explains the asymmetry of the corresponding peak [cf. Figs. 3 and 8]. For plasma, the left boundary of the SASE peak is found as $\omega \simeq 10^{-2}$, whereas in metal (silver) it is located at $\omega \simeq 0.1$.

Notice, the transverse permittivity has a non-vanishing imaginary part at small *k* as well, which results in that the ASE peak decays slower at larger $\omega$, so that the ASE peak extends almost to $\omega \simeq 1$. A combination of these tendencies produces a larger intersection of the ASE and SASE peaks, which are still well separate. Larger amplitudes of the peaks for plasma are due to a much smaller decay rate $\gamma$.

**Figure 10:**
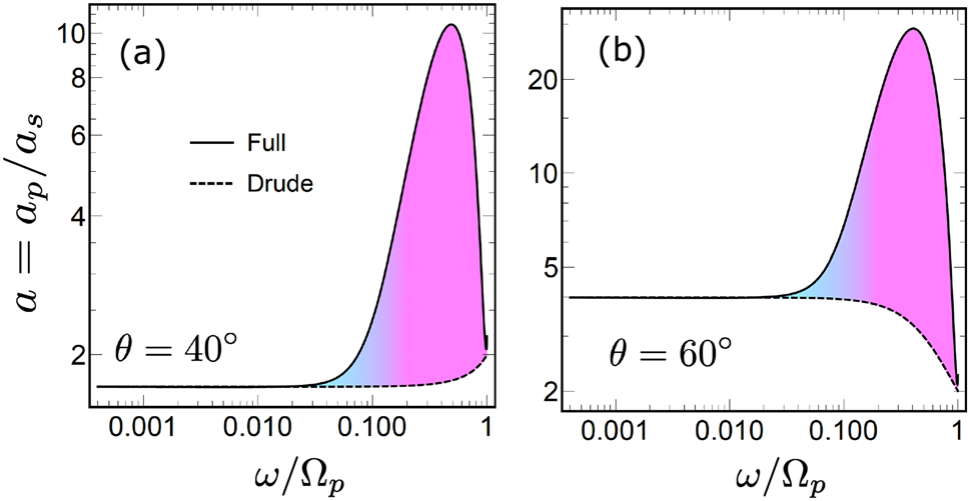
Ratio of absorptivities $a=a_p/a_s$ for *p* and *s* polarizations, calculated for plasma, for $\theta = 40^\circ$ (a), and $\theta = 60^\circ$ (b). Results for the full model in Eq. (20) (solid line ”Full”) are compared with the Drude approximation (dashed line ”Drude”).

A colour-density Fig. 9 plots the dependence of $r_p$ on the $\omega$ and $\theta$, which appears qualitatively similar to Fig. 3 for metals. Quantitative differences include a notable blue shift of the ASE peak due to increased $\alpha = v/c$ and a much larger amplitude. Finally, the absorptivity ratio *a*, plotted in Fig. 10, reveals a large SASE peak whose amplitude is sensitive to the incident angle.

## Summary and conclusion

This work reveals the relation between the superanomalous skin effect (SASE) and the enhanced absorption of light scattered on a surface of a conductive medium. The analysis is done by using an exact scattering solution for the chosen model for the dielectric response of the medium. We use the solution to extract both the SASE and the light scattering spectrum, and demonstrate that the SASE manifests itself in the additional enhancement of the light absorption in the optical frequency range $0.1< \omega /\Omega _p < 1$. The latter is well distinguished from the interval where the standard anomalous skin effect (ASE) is observed. The ASE and SASE enhancement peaks merge only in the extreme case of ultra-relativistic charge carriers.

The origin of both peaks is related to the non-locality of the dielectric response and collisionless damping of EM waves in an electrically conductive medium. However, the loss mechanisms due to SASE and ASE differ: the ASE suppresses the transverse field component, whereas the SASE affects the longitudinal one. This difference gives rise to strong dependence of the absorption spectrum on the polarization and incident angle of scattered light waves.

It is important to note that the SASE increases the energy loss even though the domain of frequencies and wave lengths where the SASE is observed is well outside those of the scattered light. The reason is that the light scattering generates a continuum of surface plasmons modes including those lying in the frequency/wave number interval where the SASE takes place. The generation of surface plasmons in the SASE domain leads to a deeper field penetration and, therefore, larger energy losses of the scattered light. The loss mechanism is related to the Landau damping where the energy of the longitudinal plasmon wave is transferred collisionlessly to the kinetic energy of charge carriers. The mechanism takes place in any electrically conductive medium. It requires the Landau damping and the longitudinal component of the electric field in the propagating EM wave. The latter is ensured by the scattering geometry when the light is scattered at a non-normal angle.

A generic origin of the loss mechanism leads us to a conclusion that the same or even larger SASE absorptivity enhancement is expected when the metal surface is not perfect. Surface irregularities randomize the scattering angle for both the EM wave and electrons in the metal. This results in a larger contribution of the longitudinal field component of ab EM wave propagating inside the metal and in a larger contribution of the waves with large *k*. These factors enhance a generation of surface plasmons leading to a larger energy loss. It is known that the light-plasmon coupling can be increased by scratching the metal surface or by placing small light scatterers in its vicinity. Also, numerical calculations using phenomenological models for the diffusive electron scattering on imperfect surfaces demonstrated only quantitative but not qualitative changes in the absorptivity spectrum^33^. However, further analysis is needed as the SASE-related effects are not accounted in those models.

Finally, we note that the visibility of the SASE peak in experiments depends on many factors, in particular, the decay rate of the carrier states. The calculations for silver demonstrate that interband transitions in the optical frequency range do not suppress the SASE peak, which remains visible at intermediate incident angles. The SASE-induced effects should, thus, reveal themselves by comparing absorption spectra for different polarization and scattering angle of the incoming light. These differences cannot be described using popular hydrodynamic models or single-pole approximations for the dielectric permittivity function.

## Supplementary Information

Supplementary Information.

## Data Availability

The datasets used during the current study are available from the corresponding author on reasonable request.
