# Supplementary figures and images for: Superanomalous skin-effect and enhanced absorption of light scattered on conductive media
Source: Sci Rep. 2023 Mar 29;13:5103. doi: 10.1038/s41598-023-31478-y (PMC10060395; doi:10.1038/s41598-023-31478-y)

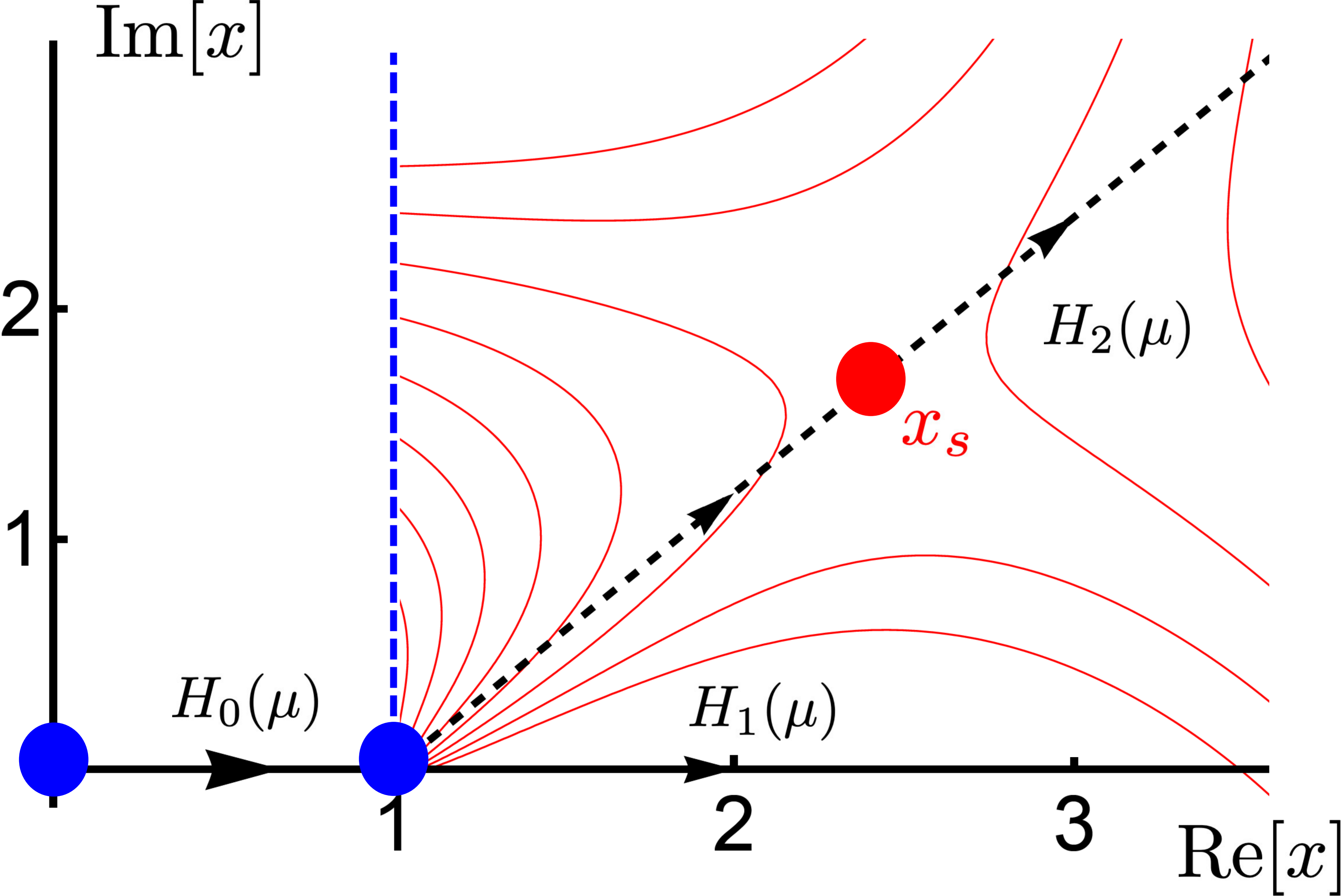

Supplement: Supplementary file 1 — Supplementary Information. [file 41598_2023_31478_MOESM1_ESM.zip › Fig11.pdf]
